# Engineering T7 bacteriophage as a potential DNA vaccine targeting delivery vector

**DOI:** 10.1186/s12985-018-0955-1

**Published:** 2018-03-20

**Authors:** Hai Xu, Xi Bao, Yiwei Wang, Yue Xu, Bihua Deng, Yu Lu, Jibo Hou

**Affiliations:** 1 Institute of Veterinary Immunology & Engineering, Jiangsu Academy of Agricultural Science, Nanjing, Jiangsu Province 210014 China; 2 Jiangsu Co-innovation Center for Prevention and Control of Important Animal Infectious Diseases and Zoonoses, Yangzhou, Jiangsu province 225009 China

**Keywords:** Bacteriophage T7, Targeting delivery, Eukaryotic expression, Vaccine

## Abstract

**Background:**

DNA delivery with bacteriophage by surface-displayed mammalian cell penetrating peptides has been reported. Although, various phages have been used to facilitate DNA transfer by surface displaying the protein transduction domain of human immunodeficiency virus type 1 Tat protein (Tat peptide), no similar study has been conducted using T7 phage.

**Methods:**

In this study, we engineeredT7 phage as a DNA targeting delivery vector to facilitate cellular internalization. We constructed recombinant T7 phages that displayed Tat peptide on their surface and carried eukaryotic expression box (EEB) as a part of their genomes (T7-EEB-Tat).

**Results:**

We demonstrated that T7 phage harboring foreign gene insertion had packaged into infective progeny phage particles. Moreover, when mammalian cells that were briefly exposed to T7-EEB-Tat, expressed a significant higher level of the marker gene with the control cells infected with the wide type phage without displaying Tat peptides.

**Conclusion:**

These data suggested that the potential of T7 phage as an effective delivery vector for DNA vaccine transfer.

## Background

DNA vaccination is the direct introduction of genetic material (containing DNA or RNA) into the host cell via injection, oral administration or particle bombardment [1]. The coding sequence of a protective antigenic gene is incorporated into the plasmid DNA, which will allow its expression in the host cells. DNA vaccination can elicit both humoral and cellular immune responses, and give protection against a variety of pathogens, tumor antigens and allergens [2, 3]. DNA vaccine potentially has several advantages over traditional vaccines, cheaper and easier to produce, have less adverse side effects, has been developing rapidly in recent years. However, the application of DNA vaccine is largely limited by its susceptibility to intercellular or extracellular endonucleases degradation. So far, several strategies have been attempted to address this issue, but none has been complete successful [4, 5].

The principle of DNA vaccine is similar to viral infection mechanisms inside the host. Both viral and non-viral vector systems have been used for DNA delivery in clinical trials [6, 7]. Superior gene delivery vectors using eukaryotic viruses including adenovirus, adeno-associated virus, retrovirus, and lentivirus have been frequently reported [8]. Also, a variety of non-viral systems have been developed, such as lipids, liposomes [9], polymers, polymersomes, cell-penetrating peptide and inorganic nanoparticles [10–14]. Bacteriophages are the most prospective biological nanomaterials, have attracted increasingly more attention as a novel DNA delivery system. Phage vectors have several advantages over viral and non-viral DNA delivery systems due to a number of promising characteristics. Bacteriophage is a nano-sized natural system capable of harboring foreign DNA insertion and efficient packaging. Most importantly, phages are safe, and have been used for treatment of bacterial infections, both in human and animals, with no safety concerns being identified [15]. Moreover, large-scale production and purification of phage particles are simple and economical. Among bacteriophages, lambda phage have been suggested as a good candidate for delivering DNA vaccine into eukaryotic cells [16, 17]. Lambda phages carrying DNA vaccine expression cassette, consisting of an eukaryotic promoter, antigen gene and polyadenylation site, can be propagated and purified for immunization against hepatitis B [18]. In addition, lambda phage particles expressing heterologous gene from eukaryotic expression cassettes have also been used for tumor therapy in a mice model [19]. Filamentous phages have been used as DNA vaccine delivery vehicle against human syncytial virus [20]. Although bacteriophages have no tropism for mammalian cells, they can be modified to display targeting ligands on the particle surface as fusion coat proteins without disrupting the phage structure [21–24]. The surface displayed targeting ligands then guide the binding and internalization of the phage particles into cells. Moreover, transfection efficacy is directly related to the copy number of the targeting ligands [25, 26].

T7 phage possesses a 55-nm diameter icosahedral head that encapsulates a 40 kb double stand DNA genome coding for 55 proteins. Under optimal conditions, T7 phages have a multiplication cycle of 11 min and produce about 10^13^ offspring particles in 1 h of replication cycle [27], and thus are suitable for large-scale production. The two main capsid proteins (10A and 10B) of T7 phages have been engineered for surface display systems that can display peptides up to about 50 amino acids in size in high copy number (415 per phage). However, T7 phage delivering genetic material has not been previously reported. In this study, we herein engineered a T7 phage as a DNA vaccine targeting delivery vector, with surface displaying Tat peptide and genome insertion eukaryotic expression box.

## Methods

### Construction of shuttle plasmids

**Fig. 1:**
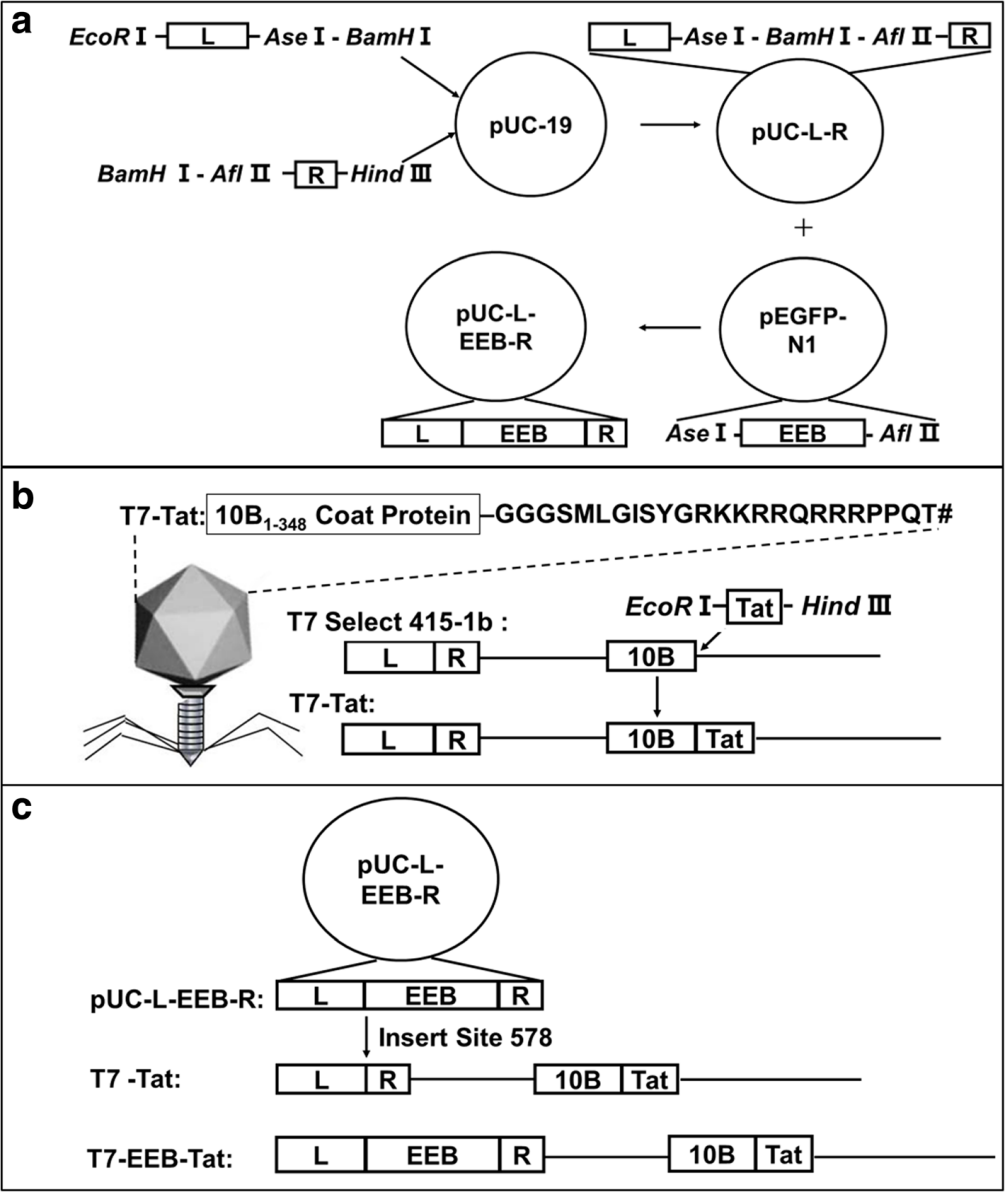
Construction of plasmids and structure of recombinant T7 phage. **a** Homologous arms were amplified by PCR and cloned into pUC-19 to construct plasmid pUC-L-R. Intact eukaryotic expression box was cloned into pUC-L-R to construct shuttle plasmid pUC-L-EEB-R. **b** *tat* gene was artificial synthesized and inserted into T7 selected 415-1b genome to rescue recombinant phage T7-Tat. **c** Recombinant phage T7-EEB-Tat was constructed which display Tat peptides on surface and contain eukaryotic expression box in the left part of its genome

Whole-genome comparison of T7 select 415-1b genome (Merck) and wild type T7 phage, about 2000 bp gene deletion would be found at position 578. PCR method was used to amplify the gene sequences L (Position: 1–578, Primer: P1-F/P578-R) and R (Position: 579–779, Primer: P579-F/P779-R) as left / right homologous recombinant arms. The fragments of L and R were inserted into pUC-19 vector using *EcoR*I/*BamH*I and *BamH*I/*Hind*III sites following the routine protocols to construct the shuttle plasmid pUC-L-R. The required restriction enzyme recognition sites *Ase*Iand *Afl*II were introduced from the primer P578-R and P579-F, respectively. Eukaryotic expression box (EEB) in plasmid pEGFP-N1 position 8–1640 was cut down with enzyme *Ase* I and *Afl* II, and inserted into shuttle plasmid to construct homologous plasmid pUC-L-EEB-R (Fig. 1a). All the primers used in this study were listed in Table 1.

**Table 1:** The primers used in this study

Primers name	Primers oligonucleotides
P1-F	AGAGAATTCTCTCACAGTGTACGGACC
P578-R	CACGGATCCCCATTAATTCGTGCGACTTATCAGGC
P579-F	CACGGATCCGGCTTAAGCAGAAAGAAATTGACCGC
P779-R	CCCAAGCTTATTGGTTCTTCCTAGTAC
T7 Select UP	GGAGCTGTCGTATTCCAGTC
T7 Select DOWN	AACCCCTCAAGACCCGTTTA
T7 558-578	CAGCCTGATAAGTCGCACGA
T7 579-599	GCGCGGTCAATTTCTTTCTG

Sequences under the line are restriction enzyme recognition site

### Construction of surface display phage

The gene sequence coding Tat peptide (MLGISYGRKKRRQRRRPPQT) with a GGGS linker was codon optimized and artificially synthesized (GenScript, Nanjing, China). The *tat* gene was cloned into T7 select 415-1b *EcoRI* / *HindIII* double-digested T7 phage genomic arms to rescue T7-Tat recombinant phage (Fig. 1b). Briefly, *tat* gene and T7 select 415-1b genome were ligated by using DNA ligation kit Ver.2.0 (Takara, Dalian, China), the ligation reaction was mixed with T7 phage packaging extract to rescue infectious phage particles. The packaging efficiency was determined by agarose double-layer plate titration by plaque assay using *E. coli* BL21 as a host. The plaques were counted and the phage titer was calculated and expressed as pfu/mL. The T7-Tat phage was selected by PCR amplification with the T7 Select UP/ DOWN primers (Table 1) which allowed amplification of the sequence surrounding the multiple cloning sites. All the experiment operation was referred to the T7 Select® system manual.

### SDS-PAGE and western blotting

T7-Tat phage particles was analyzed on 12% (*v*/v) polyacrylamide gel electrophoresis (PAGE) and Western-blotting assay [28]. Briefly, T7-Tat phage was diluted in 6× sample buffer [62.5 mM Tri-HCl (pH 6.8), 30% glycerol, 5% SDS, 0.01% bromophenol blue, 5% β-mercaptoethanol], boiled for 10 min, then analyzed on 12% SDS-PAGE gel. After electrophoresis, the gel was stained with coomassie brilliant blue (CBB) R-250. The protein was then electrotransferred to a nitrocellulose (NC) membrane and probed with horse radish peroxidase (HRP) labeled anti-T7 tag monoclonal antibody (Merck, 1:10000 dilution) and visualized using an HRP and Diaminobenzidine tetrahydrochloride (DAB) chromogenic development kit (Boster, Wuhan, China).

### Construction of phage contain EEB

The eukaryotic expression box (EEB) was inserted into T7-Tat phage genome by plasmid-phage cross-homologous recombination approach (Fig. 1c). Briefly, plasmid pUC-L-EEB-R (1 μL, 0.5 ng) was transformed into *E. coli* BL21 competent cells by using heat shock method. Then, the recombinant host cell was infected with T7-Tat and T7 select 415-1b phage at multiplicity of infection (MOI) of 0.0001–0.00001 for cross-integration, respectively. The liberated phage particles were detected using double-layer agarose assay. Then, the T7-EEB-Tat and T7-EEB phage were selected by using plaque-PCR assay with the primers T7 558–578 / T7 579–599 (Table 1) to amplify the region surrounding the insert site 578. The PCR products were sequenced (GenScript, Nanjing, China). The whole genome of T7-EEB-Tat and T7-EEB were prepared by phenol extraction method referred to T7 Select® system manual, and identified by using restriction digestion enzyme (*Swa* I, position 3805).

### Amplification and purification of T7 phages

The T7-EEB-Tat, T7-EEB and T7 select 415-1b phages were propagated in *E. coli* BL21. Briefly, 300 ml of M9LB medium was inoculated with 3 mL of an overnight culture of BL21 and incubated at 37 °C to reach a density at 600 nm (OD_600_) of 0.8–1. BL21 was infected with the phage nanoparticles above at a MOI of 0.001, and kept shaking at 37 °C more than 3 h until complete lysis of cells was observed. DNase I and RNase A (Takara, Dalian, China) were added 30 min before harvesting the phages from the culture medium. The suspension was centrifuged for 15 min at 6000 rpm (Avanti ® J-26 XPI, JLA-162500 Rotor) to separate the bacterial debris from the phage nanoparticles. 10% polyethylene glycol 8000 was added into the supernatant, then centrifuged for 20 min at 11000 rpm with the same rotor. The pellet was dissolved in 30 mL TBS buffer, followed by extracted with 0.1% Triton-114 to remove endotoxin and with an equal volume of chloroform to remove bacterial debris [29, 30]. The endotoxin residue was assayed by ToxinSensor™ Chromogenic LAL Endotoxin Assay Kit (Genscript, China), data not shown. Purified phage particles were examined under an electron microscope (ZEISS, Germany) by negative staining, using 1% uranyl acetate [31].

### Binding assay

T7 Select 415-1b, T7-Tat, T7-EEB and T7-EEB-Tat phage clones were characterized for their affinity towards eukaryotic cell by using binding assay method described as previously [32]. All cell culture was performed in a humidified 37 °C incubator with 5% CO_2_. Vero (ATCC® CCL-81™), BHK-21 (ATCC® CCL-10™), MDCK (ATCC® CCL-34™) and Marc-145 (Gift from National Research Center of Engineering and Technology for Veterinary Biologicals) cells were cultured in formulated EMEM (Eagle’s Minimum Essential Medium), supplemented with 10% fetal calf serum. Cells were seeded at 5.0 × 10^5^ cells/well, in 24-wells plate, and cultured for 12 h. The cells were washed once with medium and incubated with 500 μL of medium containing recombinant phage (1 × 10^8^ pfu) for 1 h. Unbound phages were removed by washing with Washing Buffer (0.5% BSA, 0.1% Tween 20 in EMEM) for 5 min for a total of eight washes. Cells were lysed with CHAPS (3-[(3-Cholamidoproply) dimethylammonio]-1-propanesulfonate) Lysis Buffer (2.5% CHAPS, 0.5% BSA in EMEM) for 10 min with gentle shaking on a rocker. The remaining cell lysis was then titered for phage with *E. coli* BL21. Phage recovery was calculated as the ratio of recovered phage versus the input phage as follows: Phage Recovery (100%) = Output phage / Input phage × 100%.

### Internalization assay

For the titration of internalized phage particles, the mode of internalization was studied as previous described [33]. Vero cells were cultured as mentioned above. Vero cells grown on 24-wells plate were washed, preincubated in serum-free EMEM medium for 30 min, and then treated with 10^8^ pfu of recombinant phages suspended in medium at 37 °C for 4 h. First, unbound phages were removed and cells were washed 8 times with Washing Buffer to remove any remaining unbound phage particles. Vero cell-bound phages were eluted with Elution Buffer (1% SDS) for 10 min on ice. Then, the cells were washed twice in Washing Buffer to remove remaining bound phages. The washes were designated as post-elution washing (PEW). Finally, Cells were lysed with CHAPS Lysis Buffer for 10 min with gentle shaking on a rocker. Phage in each fraction was kept and titrated with *E. coli* BL21.

### In vitro gene transfer

A standard protocol for gene transfer into cultured cells was followed. Vero cells were seeded at 5.0 × 10^5^ cells/ well, in 24-well plates, and cultured for 12 h. The cells were washed once with medium and incubated with 500 μL of medium containing T7-EEB and T7-EEB-Tat phage (1 × 10^8^ pfu), or purified T7-EEB genomic DNA (4.5 ng, 1 × 10^8^ copies) complexed with Lipofectamine 2000 (Invitrogen) for 6 h at 37 °C. The cells were washed twice with medium and then cultured for 48 h before assaying for the expression of EGFP genes. Purified phage genomic DNA complexed with Lipofectamine 2000 was set as positive control, the transfection process was carried out according to the procedures recommended by the suppliers. An untreated cell was set as negative control. The report gene EGFP was detected with fluorescence microscopy.

## Results

### Construction and identification of recombinant phage

**Fig. 2:**
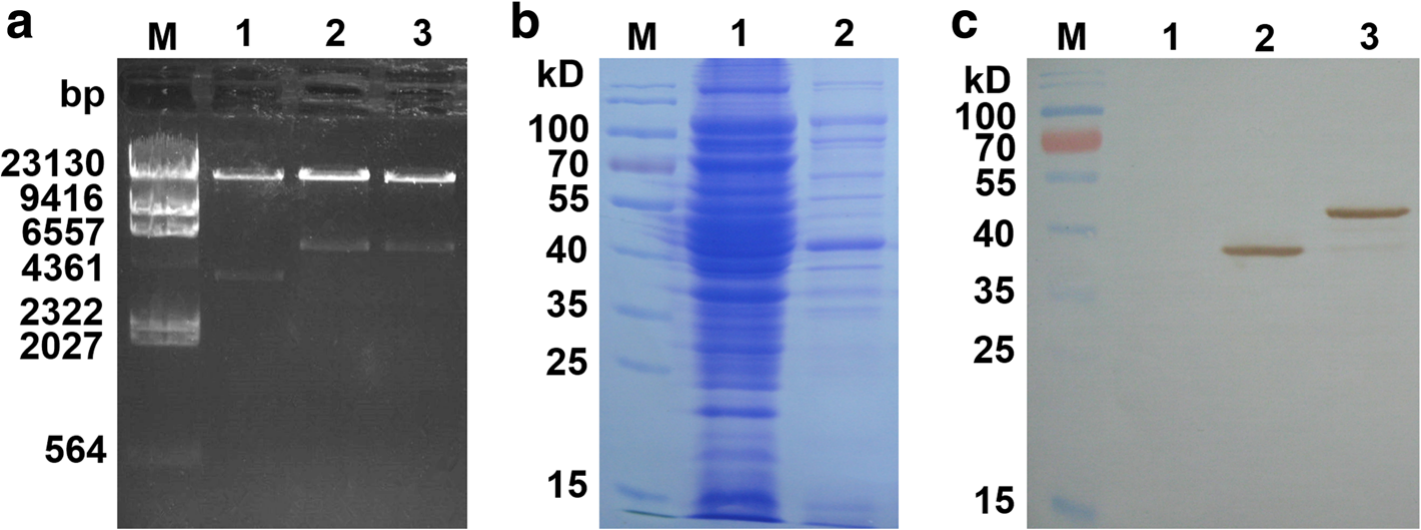
Construction and identification of recombinant phage. **a** Phage genome was digested by restriction enzyme *Swa*I. M, λ-Hind III digested molecular marker; 1, genome of T7-Tat; 2, genome of T7-EEB; 3, genome of T7-EEB-Tat. **b** SDS-PAGE on 12% polyacrylamide gel. M, protein marker; 1, lysate of T7-Tat; 2, purified T7-Tat phage. **c** Western blotting on nitrocellulose membrane using T7 Tag-HRP monoclonal antibody. M, protein marker; 1, lysate of host BL21; 2, lysate of T7 Select 415-1b; 3, lysate of T7-Tat

A high rescue efficiency of T7-Tat phage nanoparticles (4.5 × 10^5^ pfu/mL) was obtained by packaging with genomes in the included T7 protein extract. Positive clones encoding the *tat* gene fragment produced a 175 bp PCR product, and the nucleotide sequence was verified by DNA sequencing. But, T7-EEB and T7-EEB-Tat phages were generated by homologous recombinant method with a relative low efficiency, only four and six recombinant phages were identified out of fifty phage plaques. Positive clones containing EEB generate a fragment of 650 bp by PCR selection, and the PCR products were verified by DNA sequencing. High titers of T7-Tat, T7-EEB and T7-EEB-Tat phage nanoparticles (10^10^ pfu/ml) were obtained by infection of *E. coli* BL21 cultures at an MOI of 0.01 after 4 h of incubation. The above data was not shown. T7-Tat, T7-EEB and T7-EEB-Tat phage particles were concentrated, purified and the genome were extracted. The phage genomes were digested by *Swa* I, generating fragments of 3800 bp, 5300 bp and 5300 bp, respectively (Fig. 2a). Based on the length of these fragments, it was confirmed that the EEB was correctly incorporated into the left part of the T7 phage genome. The concentrated and purified T7-Tat phage were analyzed on 12% SDS-PAGE, and bacterial debris was efficiently removed (Fig. 2b). The p10B-Tat fusion protein was detected by the T7·Tag® Antibody HRP Conjugate using Western blot analysis (Fig. 2c). This observed band was consistent with the calculated molecular mass of the p10B-Tat fusion protein (40.5 kDa).

### Electron microscopy observation of T7 phages

**Fig. 3:**
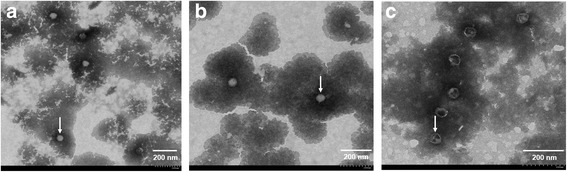
Electron microscopy observation of T7 phages. Morphology of phages (**a**) T7 select 415-1b phage. **b** T7-Tat phage. **c** T7-EEB-Tat phage. Phage particle was pointed with white arrow

The micrographs of the bacteriophage T7 was obtained by negative stained transmission electron microscopy and the phage particles was pointed with white arrow (Fig. 3). The head of T7 select 415-1b is approximately 55 nm in diameter and is connected with a very short neck. The baseplate and tail fiber cannot be clearly seen (Fig. 3a). Recombinant phage T7-Tat and T7-EEB-Tat also have an intact particle shape (Fig. 3b, c), suggesting that the insertion of a certain length of foreign gene sequences had not affected the phage particle packaging.

### Tat peptides enhance the binding and internalization efficiency of phage particle towards eukaryotic cells

**Fig. 4:**
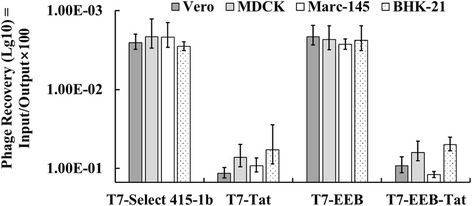
The binding efficiency of phage particle towards eukaryotic cells. T7 phages toward target cells (Vero, MDCK, Marc-145, BHK-21). Binding efficiency of the phages were estimated as their recovery (%) = output phage / input phage. Eukaryotic cells (5.0 × 10^5^ cells/ well) were incubated with 10^8^ pfu phages, thoroughly washed to remove unbound phages, and then lysed. Bacteria were infected with the cell lysates to determine output phage titer by pfu assay. Data represent mean ± S.E of 3 independent tests

**Fig. 5:**
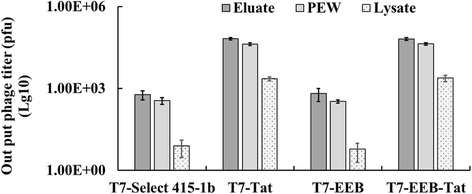
The internalization efficiency of phage particle towards Vero cells. Vero cells (5.0 × 10^5^ cells/ well) were incubated with 10^8^ pfu phages, thoroughly washed to remove unbound phages, and then the output phages were divided into Elution, PEW and Lysate. Bacteria were infected with each fraction to determine output phage titer by pfu assay. Data represent mean ± S.E of 3 independent tests

To determine whether phage surface-displayed Tat can facilitate cellular internalization of the recombinant phages, the affinity of phage particle towards the relative cell line was examined. It was found that the phage with surface display of Tat peptides can bind the eukaryotic cell more efficiently (Fig. 4). The phage recovery of T7-Tat and T7-EEB-Tat (0.05~ 0.1%) was about 15~ 30-fold higher than that of T7 select 415-1b and T7-EEB (0.003%). The affinity of Tat peptides targeting Vero and Marc-145 cell was higher than MDCK and BHK-21 cell. Furthermore, the phage particles captured by Vero cells can be divided into three fractions (Fig. 5). As expect, the output titer of T7-Tat and T7-EEB-Tat was much high than T7 select 415-1b and T7-EEB. Although, the cell surface-bound phages (Elution and PEW) took up the most percentage of total output phages, only a little of phage particles can penetrate (Lysate) into cells. The penetrate phage particles of T7-Tat and T7-EEB-Tat were more than three hundredfold over the T7 Select 415-1b and T7-EEB. The results confirmed that the presence of Tat increases the attachment rate of phages in mammalian cells in in vitro assays, which may indirectly enhance the phage penetration efficiency.

### T7-EEB-tat mediated report EGFP gene expression in cultured cells

**Fig. 6:**
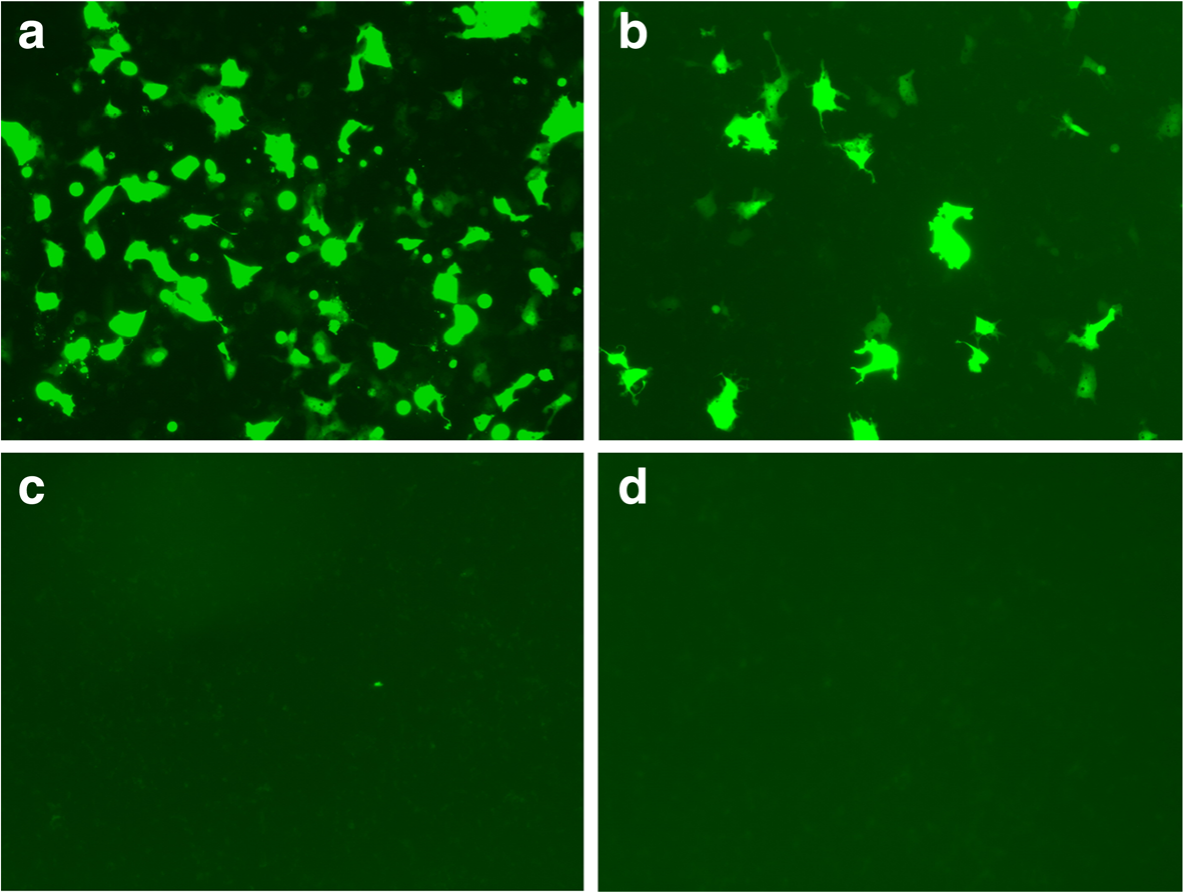
In Vitro detection of phage-mediated EGFP gene expression. Vero cells (5.0 × 10^5^ cells/ well) were incubated for 6 h at 37 °C, with 500 μl of medium containing 1 × 10^8^ pfu of phages or 4.5 ng of phage genome. **a** Purified T7-EEB genomic DNA (4.5 ng, 1 × 10^8^ copies). **b** 1 × 108 pfu of T7-EEB-Tat phage. **c** 1 × 10^8^ pfu of T7-EEB phage. **d** Untreated cell control

To identify whether recombinant T7 phage display Tat peptide could deliver EEB into cell and achieve protein expression, the T7-EEB-Tat phage was incubated with Vero cell, and the expression of the report gene EGFP was detected. After 48 h culture, the EGFP protein was detected in the cytoplasm of cells. The negative control did not express any EGFP protein, as expected (Fig. 6d). In contrast, the positive control and T7-EEB-Tat expressed a significant high content of EGFP protein (Fig. 6a, b). Because the lack of Tat peptide targeting delivery, T7-EEB did not show clear fluorescence signal (Fig. 6c). These results confirmed that the T7-EEB-Tat phage could successfully deliver EEB into eukaryotic cells and mediate the protein expression, and that the Tat peptide can facilitate the penetration of phage into cells.

## Discussion

Phage nanobiotechnology evolved from phage surface display technology has been widely used in diverse areas. Filamentous phage M13, bacteriophage T4 and λ have been used as vectors for DNA delivery into mammalian cells [34–36]. Nevertheless, the application of phage particles vector in DNA vaccine delivery is largely limited by the poor gene transfer efficiency due to the lack of eukaryotic cell targeting receptor. Previous studies have shown that the efficiency of phage mediated DNA transfer can be improved by surfacing displaying Tat targeting peptide [35, 36]. In this study, we examined the potential of T7 phage with surface-displayed Tat peptide as a DNA vaccine delivery vector. We engineered the T7 phage as a unique eukaryotic expression box delivery vector, because its size and structure resemble a condensed DNA-polymer complex. In addition, the peptide display system established using T7 phage extend its function in DNA transfer. T7 phage has several attractive characteristics that make it suitable for our experimental purpose. T7 phage particles can display various peptides as chimeras at the C terminal of the capsid protein p10 (415 copies /particle). Moreover, the genomes of T7 Select 415-1b and T7 wild type phage are different by a 2000 bp deletion at a single site, at which foreign gene can be inserted.

Due to the rapid reproductive performance of T7 phage, it is impossible to identify a recombinant phage without a selection marker. Meanwhile, the low efficiency of random homologous recombination may further increase the level of challenge. Therefore, how to effectively identify the recombinant phage that containing the eukaryotic expression box has been extremely important. The host bacterial cells were infected at a low multiplicity of infection (MOI = 0.0001–0.00001) to increase the replication cycles of the progeny phages, which contributed to yield the EEB inserted phages. T7-EEB produced smaller plaques than the T7 select 415-1b due to the replication burden of foreign gene insertion. We therefore selected the small plaques for subsequent PCR analysis. In total, four T7-EEB phages were identified out of fifty phage plaques. Further, we found that the progeny T7-EEB phage had intact particle structure, indicating that T7 phage could tolerate 2 kb insertion of foreign gene without detriment to its structure integrity. Because the foreign gene was inserted into the T7 phage genome, the loading capacity of its genome need to be further tested.

Various publications have explained the mechanism of Tat peptide in improving the transfer efficiency. Tat peptide has net positive charge, which has been proposed to enhance low affinity binding of Tat protein to the cell surface [37]. Therefore, we observed variation in the binding capacity among the different cell lines in this study (Fig. 4), which might be associated with the differences in the number of low affinity binding sites for Tat-phage. Tat peptide-mediated phage penetrate the plasma membrane is not considered to rely on endocytic pathway [38], but through caveolae [35]. By comparative analysis between the binding and internalization efficiency (Fig. 5), it was easy to find a positive correlation between it. It may indicate a possibility that Tat peptide promote phage bind with the cell surface, and then enhance the odds of phage penetrate into cell through caveolae pathway. Although the amount of Tat-phage penetrate into the cells was much higher than the phage without Tat displayed, the gene transfer efficiency mediated by Tat peptide was lower than that via Lipofectamine 2000 transfection. As shown in Fig. 6, the expression of marker gene EGFP in liposome transfection group was stronger compared with Tat-T7 phage mediate delivery. This result indicated that the liposome transfection offered advantage over the Tat-T7 phage. In this case, it would be questioned whether the design of phage mediate gene transfer is worth it. However, the Tat-T7 phage mediate internalization efficiency was about 0.002% which consistent with the previous report of Tat-M13 (0.001~ 0.005%) [36]. Considering an in vivo environment, under the protection of phage capsid protein, the effective gene content should be higher in the form of Tat phage than naked DNA. So, the next step is to switch the marker gene to an antigen gene and evaluate the potential of T7 phage as a DNA vaccine delivery vector.

## Conclusion

T7 phage is suitable for engineering and tolerancing the insertion of a certain length of foreign gene sequences in its genome. T7-EEB-Tat phage can facilitate phage targeting eukaryotic cell and achieve report gene expression. T7 phage may be potentially useful as a delivery vector for DNA vaccine transfer. The surface display capability of T7 phage also enlarge the use in vaccine design, for it can surface display antigen epitope and carry DNA vaccine within one particles. All of these possibilities remain as future challenges.
